# Combining Machine Learning Systems and Multiple Docking Simulation Packages to Improve Docking Prediction Reliability for Network Pharmacology

**DOI:** 10.1371/journal.pone.0083922

**Published:** 2013-12-31

**Authors:** Kun-Yi Hsin, Samik Ghosh, Hiroaki Kitano

**Affiliations:** 1 Okinawa Institute of Science and Technology Graduate University, Onna-son, Okinawa, Japan; 2 The Systems Biology Institute, Minato, Tokyo, Japan; 3 Laboratory for Disease Systems Modeling, RIKEN Center for Integrative Medical Sciences, Yokohama, Kanagawa, Japan; King's College, London, United Kingdom

## Abstract

Increased availability of bioinformatics resources is creating opportunities for the application of network pharmacology to predict drug effects and toxicity resulting from multi-target interactions. Here we present a high-precision computational prediction approach that combines two elaborately built machine learning systems and multiple molecular docking tools to assess binding potentials of a test compound against proteins involved in a complex molecular network. One of the two machine learning systems is a re-scoring function to evaluate binding modes generated by docking tools. The second is a binding mode selection function to identify the most predictive binding mode. Results from a series of benchmark validations and a case study show that this approach surpasses the prediction reliability of other techniques and that it also identifies either primary or off-targets of kinase inhibitors. Integrating this approach with molecular network maps makes it possible to address drug safety issues by comprehensively investigating network-dependent effects of a drug or drug candidate.

## Introduction

Drugs may interact with numerous molecules in the human body. Approximately 35% of known drugs or drug leads present multi-target activity [1]. Even when a drug is claimed to have high selectivity, it probably binds to proteins that are not identified as targets. Such unexpected off-target interactions may result in adverse reactions, which increase therapeutic risks and negatively impact drug development. An example of this is the cardiotoxicity of the tyrosine kinase inhibitor Sunitinib [2]. Concerns surrounding the use of this anti-cancer drug have arisen due to its adverse side effects. Its unanticipated inhibition of members of the ribosomal S6 kinase (RSK) and AMP-activated protein kinase (AMPK) families are at least partly responsible for the drug's cardiotoxicity [3]. Since more than two hundred proteins associated with cardiovascular diseases have been identified [4], treatment with low-selectivity drugs can have many unexpected effects. In contrast, designing drugs with multi-target therapeutic application is of increasing interest to the drug discovery community. Compared with single-target agents, drugs that regulate multiple proteins have the potential to improve the balance of efficacy and safety [5], although minimizing their toxicity remains challenging. As an example, the treatment of neurodegenerative diseases has progressed a multi-target strategy [6]. Though some multi-target drugs prove beneficial, their discovery and the identification of other clinically relevant targets is often accidental, and their final application may differ radically from their original design. Sorafenib, for example, was initially developed as a RAF kinase inhibitor, but its therapeutic contribution in curing renal and hepatocellular cancers was later ascribed to its inhibition of VEGFR2 and PDGFR, and probably other targets as well [7].

To comprehensively assess pharmacological effects, systems pharmacology has been developed [8], [9], in which various bioinformatics resources assessing different structural levels, from molecules to systems are integrated. A well-curated, comprehensive molecular interaction network is the focal point of the systems pharmacological approach. Such a network can reveal causes and effects of protein interactions over signaling networks, metabolic networks, and other related pathways. With a deeply curated network map that describes signaling cascades and interactions among molecules, one can carry out network-based screening to systematically identify target proteins of a given drug candidate and to assess its impact. Thus, network-based screening appears promising for drug repurposing and safety prediction.

Various bioinformatics resources including biological databases, signaling network construction tools, and molecular modeling software have been developed, allowing a great opportunity to meet the demands of rapid systematic screening. Given the rich data and algorithmic resources availability on one side, and urgent needs to capture poly-pharmacological effects of drugs and candidates on the other side, one obvious challenge is to develop a computational method that can accurately predict a drug's effects across molecular networks. Doing this requires development of high-precision molecular docking simulation systems, and applying them over molecular networks to compute aggregated effects of drugs.

### Issues in molecular docking simulation

Molecular virtual docking is an efficient computational method to rapidly calculate the binding potential of a small molecule, such as a drug or candidate, to a target protein. It is widely used in computer-aided drug discovery due to its speed and low cost [10]. This method is mainly used to dock a small molecule to a protein structure (i.e. pose generation) and to evaluate its potential complementarity with the defined binding site (scoring). Studies assessing the performance and accuracy of various commonly used molecular docking programs indicate that those packages are able to perform pose generation well, since most of the generated binding modes are conformationally similar to the corresponding co-crystallized ligands, but their scoring functions are still too inaccurate for a reliable prediction [11]–[13]. Plewczynski and colleagues evaluated seven popular docking programs, including Surflex [14], LigandFit [15], Glide [16], GOLD [17], FlexX [18], eHiTS [19] and AutoDock [20] on the PDBbind database [21]. The best Pearson correlations between predicted binding affinities (or scores) and experimental values were 0.38 or lower [13]. Thus, correctly predicting the binding affinity of a given protein-ligand complex continues to be one of the most challenging issues in docking simulation. Scoring algorithms such as X-Score [22] and RF-Score [23], have attempted to improve accuracy, and the best Pearson correlation value measured was 0.776 [23]. However, this correlation was obtained only in an ideal situation where binding interactions of co-crystallized complexes were directly evaluated without considering the influence of the pose prediction that is required to re-dock the native ligand to its target protein prior to scoring. When performing both pose generation and scoring function, the correlation might decrease.

In order to overcome these problems in docking simulation, multiple docking tools plus scoring functions can be applied to a given docking study to improve performance (Figure S1). This yields more than one score for each test and the best among them is identified by referring to the corresponding experimental binding affinity. Correlations can be improved from 0.61 to 0.84, depending on the tools used. However, it should be noted that the highly accurate correlation of 0.84 was achieved by manually selecting the best predictions from multiple simulators. For this approach to be practical, the best prediction from multiple simulators has to be selected automatically. To accomplish this, we developed a novel scoring approach employing two machine learning systems, which were embedded as a part of a pipeline implementing a network-based screening approach that integrates curated signaling networks, bioinformatics databases, and molecular docking simulation to comprehensively and rapidly evaluate potential binding affinities of given drugs against proteins involved in a signaling network.

## Results

### Machine learning systems for improving docking simulation

The first machine learning system we employed (A) was a re-scoring function developed to assess binding modes generated by docking tools and to rank them accordingly. Machine learning system B was a binding mode selection function designed to identify the most predictive binding mode from those originated in the previous step. A test case for these two systems is illustrated in Figure S2. Both systems were built and validated by using the PDBbind version 2007 refined set which contains 1300 protein-ligand complexes and is considered a high-quality standard dataset for theoretical studies on molecular recognition.

#### 1. Machine learning system A: a re-scoring function

Reliability of docking simulations depends upon performance of the scoring function. A recent developed method known as RF-Score [23] allows better predictions compared with other scoring functions. RF-Score applies a non-parametric machine learning algorithm called Random Forest [24] to predict protein-ligand binding affinity by assessing the number of occurrences of various protein-ligand interatomic contacts (Supplementary Table S1) within a specified distance. We adopted and further revised RF-Score in this work, so that we not only considered intermolecular interactions, but also included the quantitative structure–activity relationship (QSAR) in the machine learning model in order to extend its modeling assumptions. Molecular physicochemical properties of test compounds were parameterized as predictors in the modeling exercise (Table S2).

#### 2. Machine learning system B: a binding mode selection function

Following the re-scoring function, the second machine learning system developed in this work was a binding mode selection function, designed to assess binding modes and to identify the best predictor (Figure S3). This learning system used a multinomial logistic regression method [25]. It employed supervised learning algorithm capable of predicting probabilities of categorical placement among more than two discrete outcomes, based on a set of independent variables. Similar to binary logistic regression, multinomial logistic regression uses maximum likelihood estimation to calculate the probability of categorical outcomes and allows different types of independent variables in building a model. In the present study, independent variables were the same as the predictors used in machine learning system A. Predicted categorical outcomes include the three top-score binding modes generated by docking tools, including eHiTS, GOLD and AutoDock VINA [26]. Through the binding mode selection function, molecular interactions of the three binding modes together with molecular properties of the test compound were assessed, and finally one of them was selected which was predicted as most reliable for a particular docking study.

#### 3. Validation using PDBbind benchmark

The performance of screening with these two machine learning systems was validated on the demanding PDBbind benchmark. For testing with less bias, the validation was conducted with a re-docking experiment (Materials and Methods). Of the 1,300 complexes, 195 structures (15%) were randomly selected as the test set and remaining 1,105 structures were employed as the training set (85%). The training process with the random test/training partition was iterated 25 times in order to comprehensively assess the effects of varying the dataset. Pearson correlations between the predicted scores and corresponding experimental binding affinities were measured.

**Figure 1:**
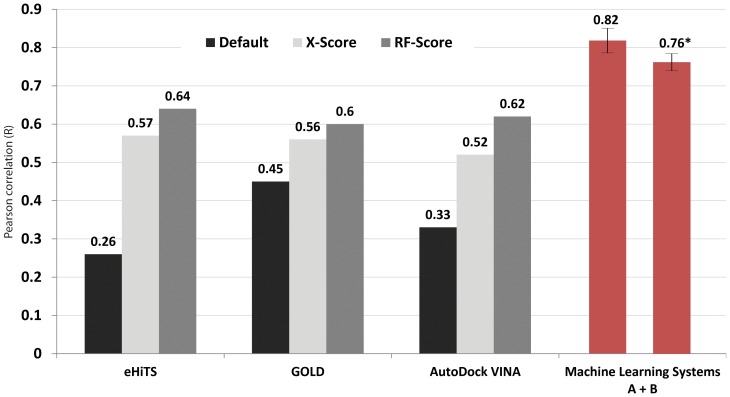
Comparison of prediction accuracy using different docking approaches. Validation data included the 1300 protein-ligand complexes of PDBbind version 2007. Values were the correlations between calculated docking scores and corresponding experimentally determined binding affinities. Black bars indicate results using default scoring functions equipped with docking tools. Gray bars are those re-scored with external scoring functions (e.g. X-Score and RF-Score) after docking. Red bars represent averages of 25 random test/training partition tests using machine learning systems A + B, and the one with an asterisk is the test using PDBbind version 2012 (2897 complexes) dataset. Error bars  =  ± one s.d. External re-scoring functions improved the correlations compared with the employment of docking simulations alone. The application of machine learning systems A + B was the most effective.

Application of external re-scoring functions (i.e. X-Score and RF-Score) improved correlations compared with the employment of docking simulations alone using the default functions equipped in the docking tools (Figure 1). Application of machine learning systems A + B was the most effective (R = 0.82, average of 25 tests). When the benchmark data were replaced with the newer PDBbind version 2012, refined set (2,897 complexes) with the same validation procedure, machine learning systems A + B also showed a strong correlation (average R = 0.76). For more extensive testing, we applied machine learning systems built with a training set (1,105 complexes from PDBbind 2007) to predict a larger test set composed of 1,792 new complexes found in PDBbind 2012. Unlike the previous validations with a test/training partition of 15%, the ratio in this test increased to 162% (1,792 V.S. 1,105). Not surprisingly, the average correlation (R = 0.65) was not as strong as observed in the previous validation, but it still presents a competitive performance compared with other methods [13]. These results indicate that the application of multiple docking tools together with the predictive machine learning systems is capable of estimating the ligand binding strength better than the use of a single docking tool and other scoring functions. Some of the consensus methods [27]–[29] apply multiple docking tools and combine the reported scores through weighting or normalizing methods for the consensus procedure. Unlike those methods, our screening approach reports a score which is a negative logarithm of experimental dissociation/inhibition constant value (p*K*d/p*K*i) usually ranging from 0 to 10 (i.e. from weak to strong binding), allowing a straightforward indication of binding strength.

The re-scoring function (i.e. machine learning system A) is essentially a learning model built upon a training set from co-crystallized complexes in PDBbind with a range of experimentally determined binding affinities. The learning model was capable of predicting the strength of binding, but was not necessarily able to recognize a compound's activity (i.e. bound or not bound to a target protein), because information about unbound compounds (inactive) was absent from the training data. In order to introduce information about inactive substances into the learning model, we added a substantial number of dummy entries to the training set, which are the binding modes of a set of test compounds against various proteins (Tables S3 and Table S4) generated by docking tools. While the test compounds were experimentally confirmed as inactive against target proteins, their binding affinity values (p*K*d/p*K*i) were set to 1. Consequently, the machine learning model contained information on both groups of compounds (i.e. active and inactive).

### A case study of target identification for kinase inhibitors

Karaman et al. proposed a quantitative analysis of kinase inhibitor selectivity against a substantial number of kinases using an *in-vitro* competition binding assay [30]. Because that study analyzed global observed interaction patterns of numerous compounds against diverse kinases, it was of great interest to assess the consistency between bioassay results and our predictive approach. Considering structure availability and binding site certainty, we selectively downloaded a set of co-crystallized kinase structures from the PDB database [31] (Materials and Methods). Finally, we selected 139 different kinases in 8 kinase groups for docking simulations (Table S5). Tested compounds include 33 kinase inhibitors interacting with various primary targets (Table S3). Karaman et al. proposed the calculation of a selectivity score (S) for each test compound, dividing the number of kinases interacting with a dissociation constant <3 µM by the number of kinases tested. A lower selectivity score indicates that a compound only interacts with a small number of target proteins, implying a lower potential for off-target effects. This constant (3 µM) is equal to a docking score 5.52 p*K*d using a negative logarithmic calculation, so we set 5.52 p*K*d as our cutoff value to “predicted selectivity score (S)  =  number of kinases docked with score >5.52/total number of kinases tested”. For example, if a test compound is docked to 100 different target proteins and 45 have a docking score >5.52, its selectivity score is 0.45.

**Figure 2:**
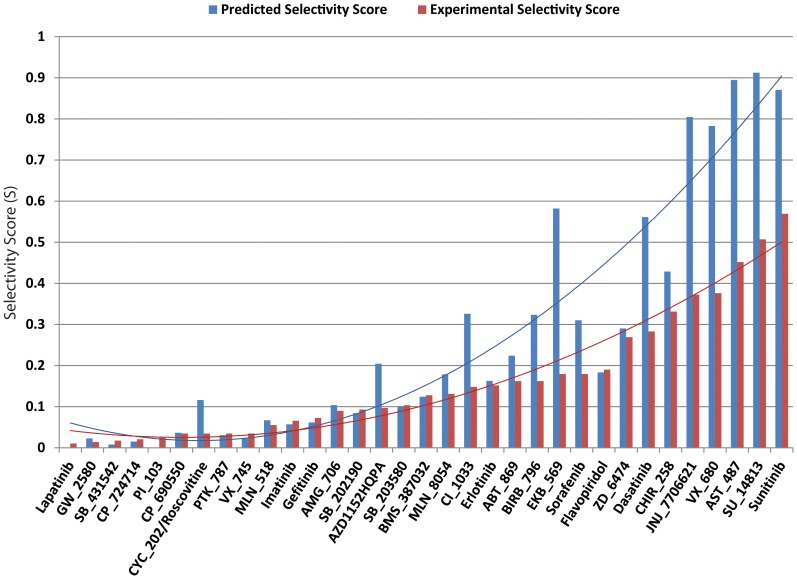
Selectivity scores of 33 kinase inhibitors against 139 kinases. A comparison was conducted using the screening approach proposed in this study (blue bars; PDB IDs from Table S5) and bioassay results [30] (red bars). The calculation of a predicted selectivity score is “S  =  number of kinases docked with score p*K*d >5.52/total number of kinases tested”, whereas the experimental selectivity scores is “S  =  number of kinases found to bind with *K*d <3 µM/number of kinases tested”. A compound with a lower selectivity score indicates that it actively interacts with a small number of target proteins, implying a lower potential for off-target effects. Trendlines are the 2^nd^ order polynomial regression functions. In most cases, screening accurately predicted the actual calculated binding constants; however, in some cases, screening predicted significantly higher binding constants than experimental data revealed, while no significant underestimates were observed.

**Figure 3:**
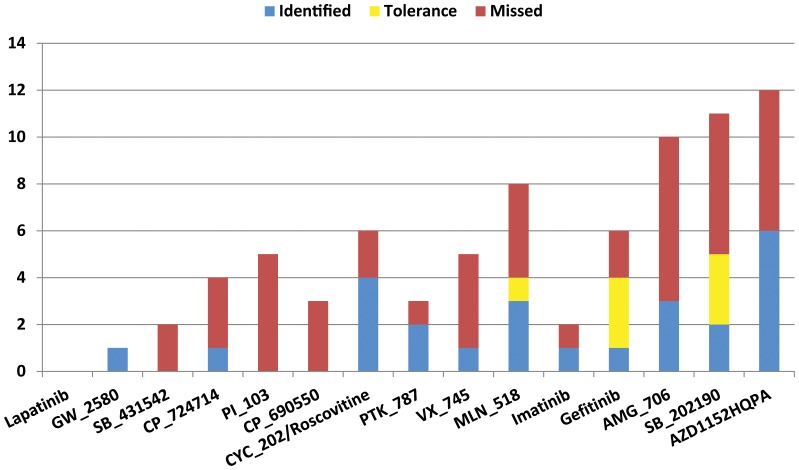
Performance of screening in identifying potential off-targets of 15 high selectivity kinase inhibitors (experimental selectivity score S <0.1). Off-target proteins are those other than the primary targets that interact with inhibitors with a binding affinity <3 µM (Karaman et al. [30]). Blue bars are off-targets that the screening approach succeeded in finding (docking score >5.52; 25 out of 72 off-targets found). Yellow bars indicate those with a tolerance (docking score >4.52; 7), whereas red bars indicate failure of the screening approach to locate any off-target proteins (46 in total).

The majority of the predicted selectivity scores were similar to experimentally measured values (Figure 2), though the screening approach tended to overestimate binding affinity in some cases. Some of the predicted selectivity scores are fairly high compared with the referred bioassay. The docking scores of each compound against the kinases are tabulated in Table S6. The screening approach succeeded in identifying a half of the primary target proteins (50% of 50), and when a tolerance is given (docking score >4.52 as cutoff value for calculating selectivity score) it identified 68% of them. Figure 3 shows the performance of the 15 high-selectivity inhibitors in identifying the off-targets. Off-target proteins are proteins other than primary targets that interacted with an inhibitor with a binding affinity <3 µM (Karaman et al. [30]). The screening approach was able to recognize one or more off-targets for the most of inhibitors, and was only clearly unsatisfactory in the cases of compounds SB_431542, PI_103, and CP_690550. For instance, compound GW-2580 was originally designed to interact with CSF1R kinase (*K*d = 1.6 nM in bioassay). Through screening, a potential off-target protein known as TRKB was suggested (docking score = 6.03) and its bioassay also indicated a stronger binding affinity (*K*d = 36 nM). Similarly, the docking simulation revealed that EGFR is also a likely off-target for CP_724714 (docking score = 8.11 and experimental *K*d = 42 nM). Overall, the screening approach succeeded in finding more than 32% of off-targets (25 out of 78) of the 15 selective inhibitors, and this score was improved (∼41%) when a tolerance was given.

### Integrating into a network-based screening system

**Figure 4:**
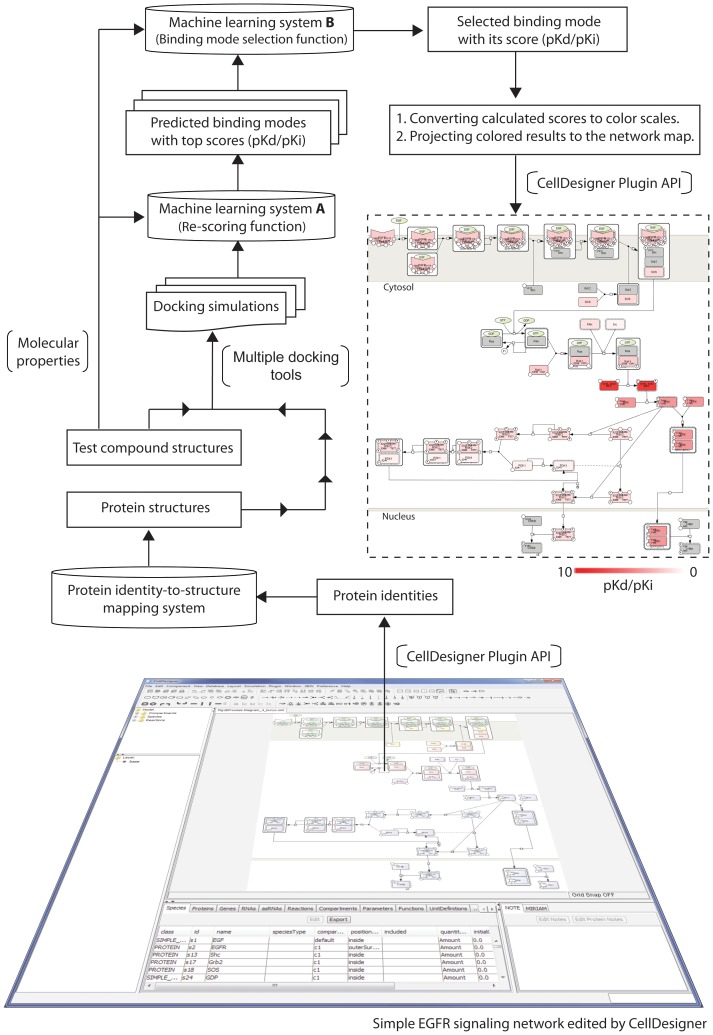
Schematic of the signaling network-based screening pipeline. First, a signaling network is launched by CellDesigner. The identities of proteins involved in the network are retrieved by the CellDesigner plugin API to look up corresponding protein structures in 3D through a protein identity-to-structure mapping system. Second, users submit test compounds for docking simulation. After docking simulation using three docking tools, machine learning system A is then applied to re-score generated binding modes based on features of binding interactions and the test compound's molecular properties, after which, it ranks them. Machine learning system B is subsequently to select a binding mode with the greatest reliability from the three top-score modes. Screening is iterated to assess the next protein until all pathway proteins have been tested. Finally, docking scores are converted into a white-to-red color scale to interpret binding strength, and are projected on the network map for a comprehensive inspection.

The system described in this article is a plug-in to CellDesigner [32], enabling molecular docking simulations to be performed with specified molecules in a network loaded to CellDesigner (Figure 4). CellDesigner is one of the most widely used graphical editors for deep curation [8] and is capable of capturing a large-scale signaling network consisting of more than a thousand molecular species and reactions [33].

For the sake of simplicity, we used a simple EGFR signaling network edited by CellDesigner as an example, although a large-scale comprehensive network would be used for real cases. The EGFR network describes signaling cascades of 14 different proteins with 27 known reactions (Figure S4). Identities of the proteins (i.e. protein names) shown on the network map were first retrieved by the CellDesigner plugin API, to look up the referred protein structures in 3D through a protein identity-to-structure mapping system. Protein identity information stored in the mapping system was mainly obtained from EMBL-EBI [34], and 3D structures deposited were from the PDB database. Through the plug-in GUI interface, users can quickly select structures of proteins listed on the network for large-scale screening. By default, the protein binding site for subsequent docking simulation is automatically defined as that to which the biggest native ligand of the co-crystallized complex binds. If only an orphan protein structure is available or an alternative site is preferred, users can easily specify another site through the plug-in. Users can upload a reasonable number of test compound structures in 1D, 2D, or 3D, and the screening pipeline accepts most common formats, such as MDL SDF [35], Sybyl Mol2 [36] and Daylight SMILES [37]. Alternatively, users can compose a compound structure manually using a built-in chemical editor [38].

Following structure file preparation, docking simulation is then carried out using multiple docking tools, including eHiTS, GOLD, and AutoDock VINA. Machine learning systems A + B were then applied to evaluate the binding potential between the test compound and a protein. Screening was iterated to assess subsequent proteins until all of the pathway proteins have been tested. Subsequently, docking scores, that is, the predicted binding affinities of the test compound against all of the network proteins, are converted into a white-to-red color scale from 0 to 10 (p*K*d/p*K*i; see Figure 4). A docking score that exceeds the range is set as 10. Colored results are then projected to the network map to directly display predicted binding affinities. Proteins in gray indicate cases in which 3D structures were not available, or none of the binding modes is generated by the docking tools. Through the colored network map generated by the system, users can efficiently investigate potential bioactivity of a test compounds against numerous proteins and can also carry out a perspective inspection for the results throughout a complex signaling network. For example, a test compound had a strong interaction with MEK protein. As a consequence, not only MEK might be functionally impaired, but the phosphorylation of all downstream target molecules (e.g. ERK and RSK2) would be inhibited, negatively impacting regulation of the cell cycle. Blocking MEK-ERK-RSK2 signaling results in reduced activation of several transcriptional regulators of proliferative genes in tumor cells (e.g. melanomas and myeloma) and suppresses cell growth [39]. In addition, side effects, such as dermatologic toxicity caused by MEK inhibitors, have been reported because inhibition of MAPK signaling causes decreased cell migration, keratinocyte cell death, and inflammation [40]. Since Figure S4 presents a very simple pathway map, it does not show possible interactions with proteins in other pathways. However, the use of larger, more comprehensive maps for the EGFR pathways [41], Toll-Like Receptor pathways[42] and mTOR pathways [33], or maps developed for specific biological processes related to potential side-effects, would uncover such risks.

## Discussion

Docking simulation, a screening method to rapidly assess a test compound's binding activity, is especially helpful in early stage pharmacology studies. We developed a docking method using machine learning approach, which integrates features of structure-based rational drug design and QSAR into the learning models to enhance performance in molecular recognition. Docking simulation conducted by machine learning systems A + B provides improved reliability in predicting binding potentials and the capability of identifying potential targets. To achieve more accurate prediction, further integration of other computer-aided technology is feasible, such as the application of molecular dynamics (MD) after docking. Together with a curated signaling map, the network-based screening approach is able to comprehensively characterize the underlying mechanism of a drug candidate's activity and also to interpret the cascade effects of modulated targets. Adverse side effects constitute an enormous cost in drug development. By applying network-based screening, drug developers can reduce the possibility of marketing a drug with unfavorable drug-target interactions. On the other hand, it also provides an opportunity to rationally optimize inhibitor polypharmacology for treating complex diseases, such as cancer, neurodegenerative disorders, cardiovascular disease, and metabolic syndromes.

## Materials and Methods

### Re-docking experiment for model validation

1. Molecular structure files: Protein-ligand complex files for re-docking experiments were obtained from the PDBbind database. To validate predictive models with less bias, native ligands of the co-crystallized complexes were first extracted and converted into 2D using Open Babel [43]. For the following docking simulation, 2D structures were then re-converted to 3D using a 3D structure generator called CORINA version 3.4 [44].

2. Molecular docking simulation packages: Native ligands were docked to their corresponding target proteins using eHiTS, GOLD, and AutoDock VINA (Table S7). These docking tools are used to generate numerous binding modes of the test compound in a defined binding site, and the number of binding modes generated varies with the docking tools. For a docking simulation, eHiTS was set to output 1000 conformations for each docking study. Considering the computing speed of GOLD, we set the maximum as 300. The maximum binding mode of AutoDock VINA varies with an energy range of 10 (kcal/mol).

3. Application of machine learning systems: Binding modes generated by the three docking tools were re-scored by machine learning system A, and only the three top-score candidates in each set were retained. Subsequently, machine learning system B assessed the three top-score candidates and identified the most predictive one. Modeling exercises of the machine learning systems A and B were conducted using the R statistical package. The Random Forest algorithm was applied to build machine learning system A, which was implemented in “randomForest” (Breiman and Cutler's random forests for classification and regression) module. For machine learning system B, the multinomial logistic regression of “nnet” (Feed-forward Neural Networks and Multinomial Log-Linear Models) and “MASS” (Modern Applied Statistics with S. Fourth Edition) modules was utilized.

4. Re-docking result: The Pearson correlation coefficient between the predicted docking scores and the experimental binding affinities was calculated using R to determine the predictiveness of the screening approach.

### Case study of target identification for kinase inhibitors

1. Protein structure files: protein structures collected from the PDB database complied with the following criteria: 1) X-ray structures with resolution of 2.5Å or better, if available 2) if two or more structures were available, that with the best solution was selected 3) a structure with a ligand bound to its nucleotide binding site was selected 4) non-modified and non-phosphorylated residues found in the binding site were selected with priority 5) the organism was human.

2. Test compound files: test compound structure files in 2D format were downloaded from PubChem, and converted into 3D using CORINA version 3.4 for the docking simulation.

3. Molecular docking simulation: the use of the docking tools was the same as mentioned in the re-docking experiment.

## Supporting Information

Figure S1Performance of docking simulations applying multiple docking tools and scoring functions to the PDBbind database in order of measured Pearson correlations between docking scores and experimental binding affinities. Four docking programs and two scoring functions were paired to form a set of unique combinations (at least three pairs in each combination): 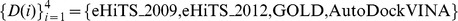


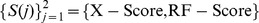


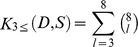
where *D* and *S* were docking programs and scoring functions, respectively. *K_3≤_* (*D*,*S*) represents the sum of all possible unique combinations, in each of which the number of paired tools varied from three to eight. There were 219 unique combinations in total. In docking tests, each of the native ligands was re-docked to its target proteins using individual docking programs and re-scored with the scoring functions. A best score in every docking study was then identified manually, which was closest to the corresponding experimental binding value. As a result, the one uses eight paired tools can give a best correlation (R = 0.84), whereas the lowest is 0.61 while only three paired tools (E_F_G) are used.(TIF)

Figure S2 Use of two machine learning systems in a docking study. A test compound is firstly docked to the target protein using three docking tools. Three sets of binding modes are generated by these docking tools and the number of binding modes is varied by the docking tools (eHiTS: 1000; GOLD: 300; VINA: ≤1000). According to the features of binding interactions (36 atomic contacts) and the test compound's molecular properties (74 descriptors), machine learning system A rescores and ranks all of the binding modes. Only the top-score binding mode in each set is kept. Afterward, based on the characterized binding interactions and molecular properties, machine learning system B is then applied to calculate the probabilities for the three top-score binding modes. The mode with highest probability is considered the most reliable for this docking study. In this case the binding mode generated by GOLD with its score is predicted to be the closest to the corresponding experimental binding affinity.
(TIF)

Figure S3 Performance of machine learning system B in identifying the most predictive binding modes in order of measured success rate. PDBbind complex structures are used to perform the re-docking experiment using the tools mentioned in Figure S1. There were 219 unique combinations in total. In a re-docking experiment, a native ligand was re-docked to the target protein using different tools. The machine learning system was to assess the generated binding modes and to eventually select one of them. It was defined as a successful prediction when the docking score of the selected mode were closest to the corresponding experimental binding affinity. The black solid line is the success rate using the machine learning system, whereas the gray dashed line represents the result using random selection as a contrast. Given the obvious difference between the results, the machine learning approach is clearly capable of identifying the most predictive binding mode for a particular docking study.
(TIF)

Figure S4 Simple EGFR signaling network edited by CellDesigner using SBGN (Systems Biology Graphical Notation). From the binding of EGF to EGFR on cell membrane to the catalysis of CREB and c-Myc within nucleus, there are 14 different proteins with 27 known reactions on the map. Upon recruitment of FGR-FGFR-Shc-Grb2-SOS complex, binding of GTP to Ras is induced, followed by formation of the GTP-Ras-Raf1 complex. Phosphorylation of the GTP-Ras-Raf1 complex is catalyzed by PAK and Src, leading to a series of subsequent phosphorylations of MEK, ERK and others.
(TIF)

Table S1 Interaction types of the 36 interatomic contacts used in the development of both machine learning systems A and B. Contacts of atoms (C, N, O, F, P, S, Cl, Br and I) between the ligand and protein within a distance of 12 Å were counted. There were 81 different atom pairs, of which 45 were omitted in this study because none of PDBbind complexes contains F, P, Cl, Br or I atoms. As an example, C_C indicates the interaction type in which carbon atoms of a ligand interact with protein carbon atoms within a 12 Å radius. The number of occurrences of this interaction was counted.
(DOCX)

Table S2 Descriptions of the 74 molecular physicochemical properties used in the development of machine learning systems A and B. There were separated into six groups. These molecular properties were calculated using the Dragon software package (http://www.talete.mi.it/).
(DOCX)

Table S3 Compounds composing the training set for building the re-scoring function of machine learning system A. Chemical figures were obtained from PubChem website, and information about primary targets came from the work of Karaman et al.^30^.
(DOCX)

Table S4 Proteins for generating binding modes that composed the training set comprising the re-scoring function of machine learning system A.
(DOCX)

Table S5 Kinase proteins for the case study using the screening approach proposed in present work. There were 139 different kinase structures covering eight kinase groups in total.
(DOCX)

Table S6 The docking scores of each compound against 139 kinases.
(XLSX)

Table S7 Parameters and settings for the docking simulation in this work.
(DOCX)
