# Supplementary figures and images for: Combining Machine Learning Systems and Multiple Docking Simulation Packages to Improve Docking Prediction Reliability for Network Pharmacology
Source: PLoS One. 2013 Dec 31;8(12):e83922. doi: 10.1371/journal.pone.0083922 (PMC3877102; doi:10.1371/journal.pone.0083922)

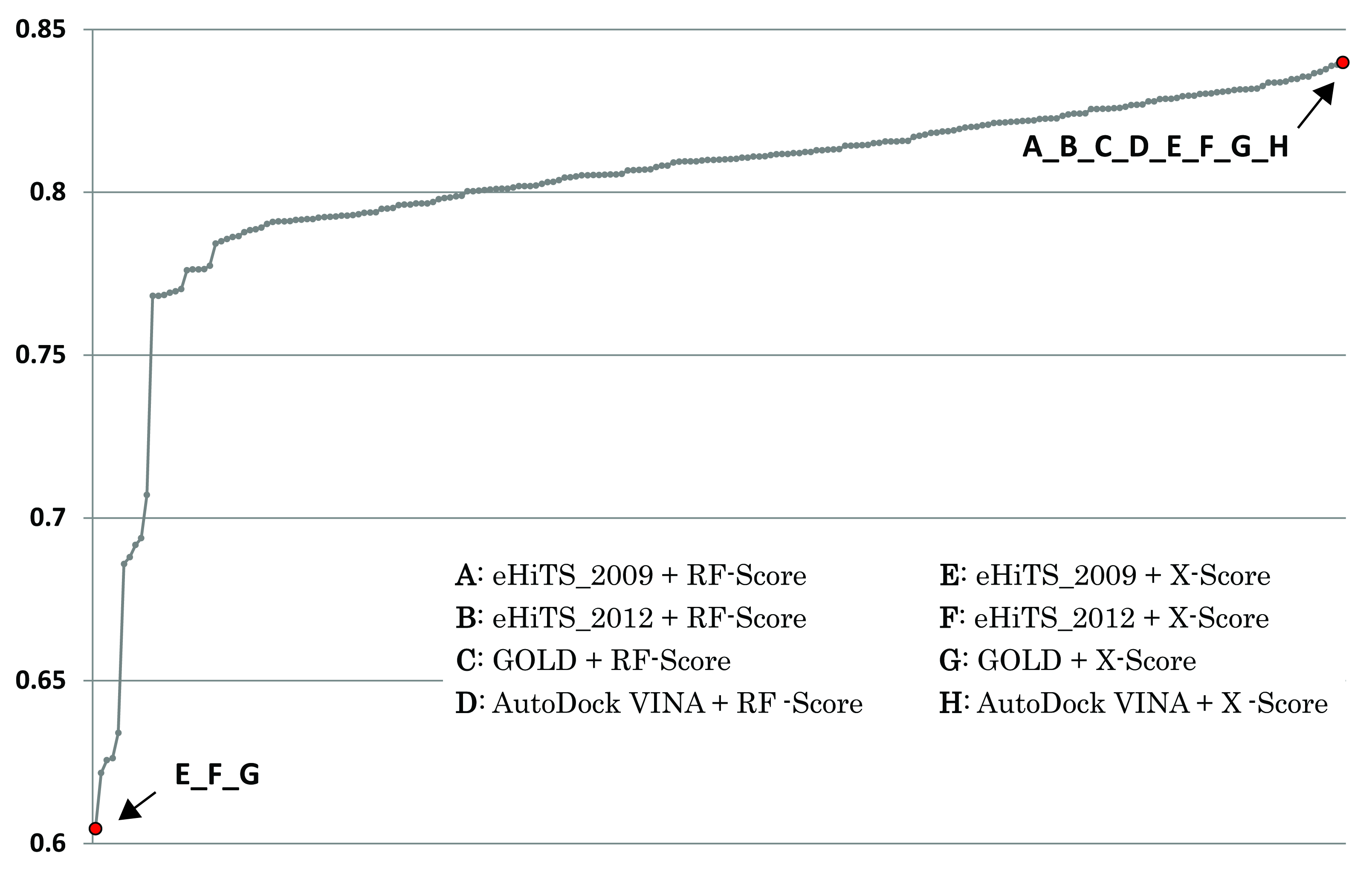

Supplement: Figure S1 — Performance of docking simulations applying multiple docking tools and scoring functions to the PDBbind database in order of measured Pearson correlations between docking scores and experimental binding affinities. Four docking programs and two scoring functions were paired to form a set of unique combinations (at least three pairs in each combination): where D and S were docking programs and scoring functions, respectively. K3≤ (D,S) represents the sum of all possible unique combinations, in each of which the number of paired tools varied from three to eight. There were 219 unique combinations in total. In docking tests, each of the native ligands was re-docked to its target proteins using individual docking programs and re-scored with the scoring functions. A best score in every docking study was then identified manually, which was closest to the corresponding experimental binding value. As a result, the one uses eight paired tools can give a best correlation (R = 0.84), whereas the lowest is 0.61 while only three paired tools (E_F_G) are used. (TIF) [file pone.0083922.s001.tif]

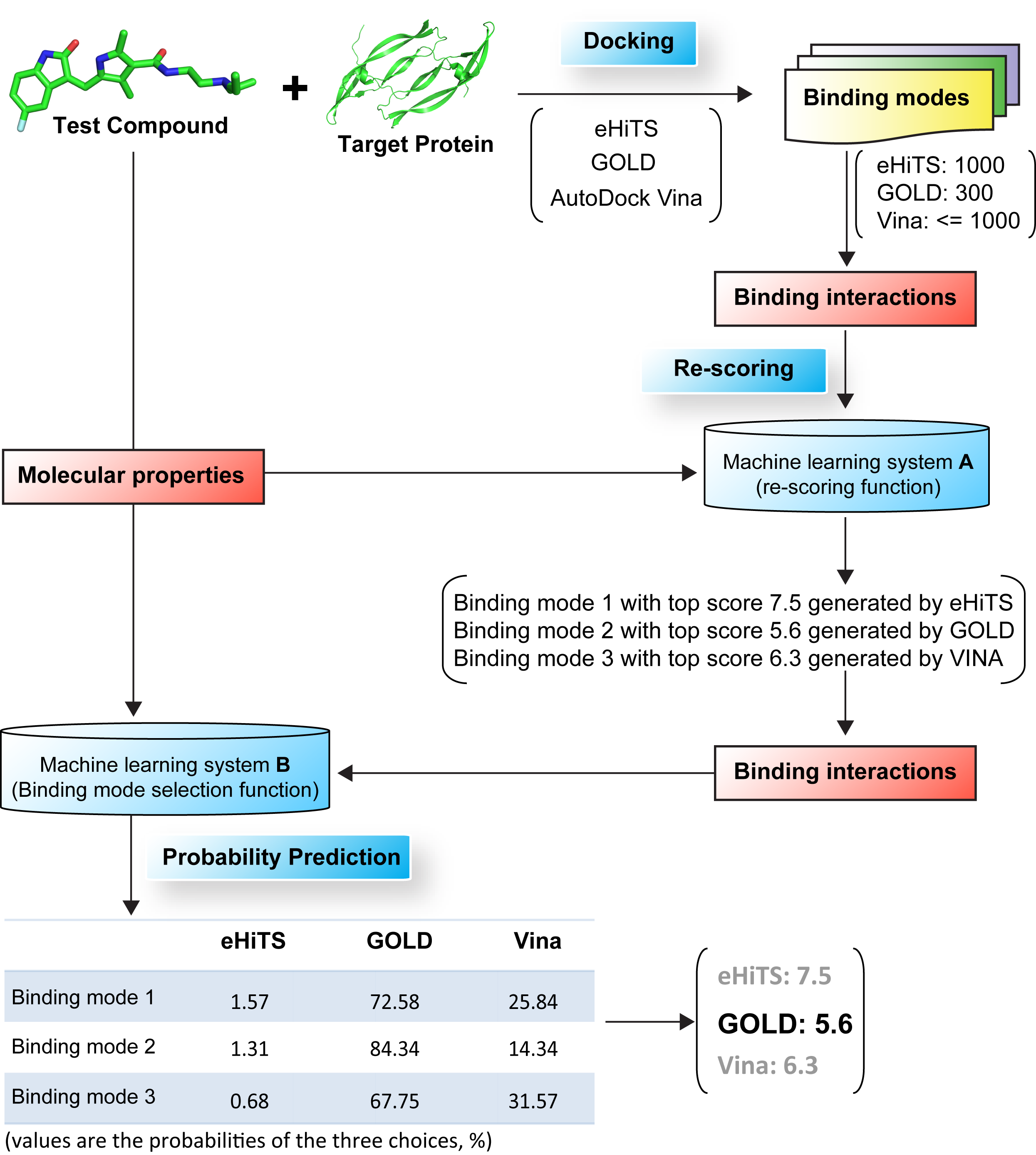

Supplement: Figure S2 — Use of two machine learning systems in a docking study. A test compound is firstly docked to the target protein using three docking tools. Three sets of binding modes are generated by these docking tools and the number of binding modes is varied by the docking tools (eHiTS: 1000; GOLD: 300; VINA: ≤1000). According to the features of binding interactions (36 atomic contacts) and the test compound's molecular properties (74 descriptors), machine learning system A rescores and ranks all of the binding modes. Only the top-score binding mode in each set is kept. Afterward, based on the characterized binding interactions and molecular properties, machine learning system B is then applied to calculate the probabilities for the three top-score binding modes. The mode with highest probability is considered the most reliable for this docking study. In this case the binding mode generated by GOLD with its score is predicted to be the closest to the corresponding experimental binding affinity. (TIF) [file pone.0083922.s002.tif]

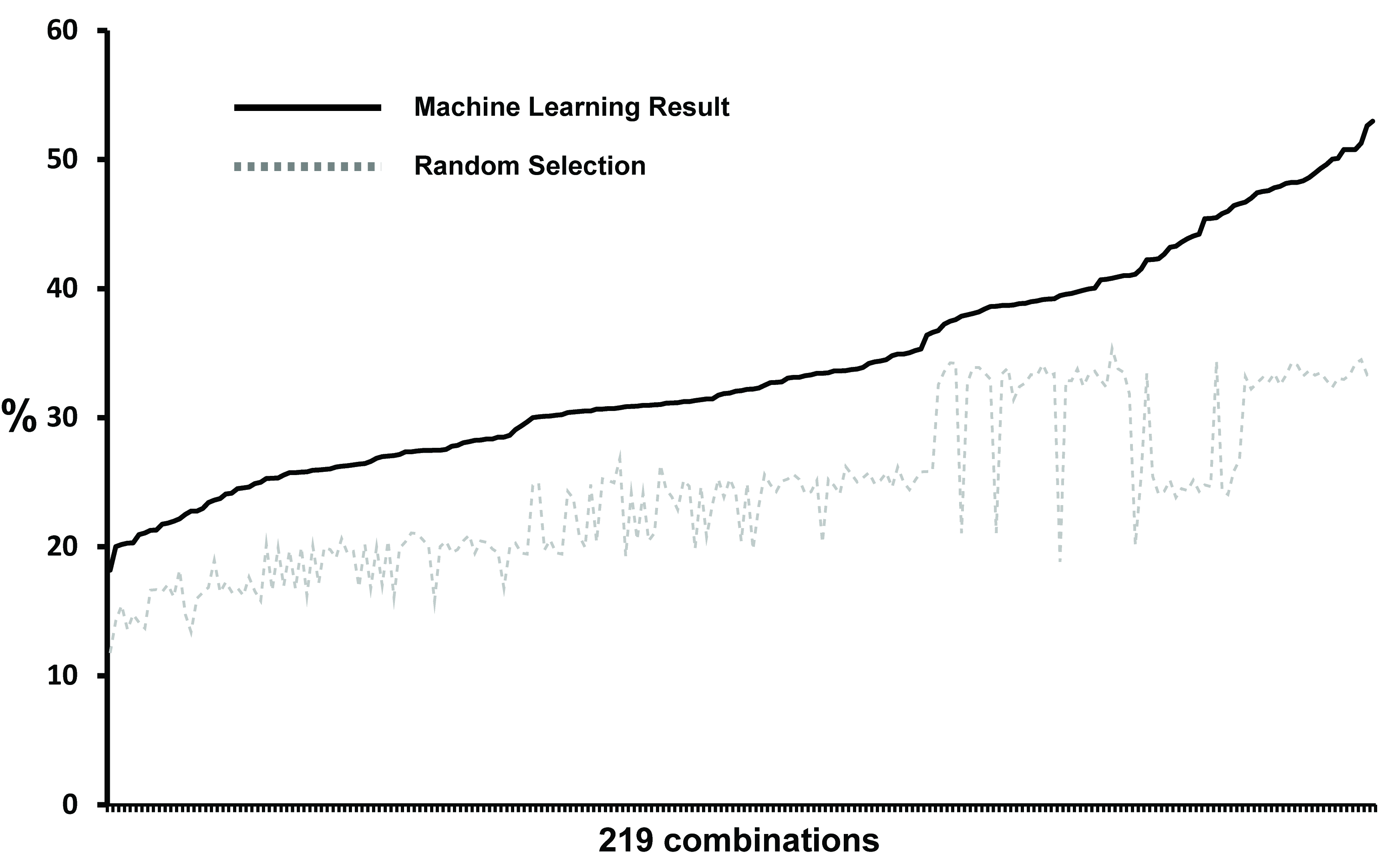

Supplement: Figure S3 — Performance of machine learning system B in identifying the most predictive binding modes in order of measured success rate. PDBbind complex structures are used to perform the re-docking experiment using the tools mentioned in Figure S1. There were 219 unique combinations in total. In a re-docking experiment, a native ligand was re-docked to the target protein using different tools. The machine learning system was to assess the generated binding modes and to eventually select one of them. It was defined as a successful prediction when the docking score of the selected mode were closest to the corresponding experimental binding affinity. The black solid line is the success rate using the machine learning system, whereas the gray dashed line represents the result using random selection as a contrast. Given the obvious difference between the results, the machine learning approach is clearly capable of identifying the most predictive binding mode for a particular docking study. (TIF) [file pone.0083922.s003.tif]

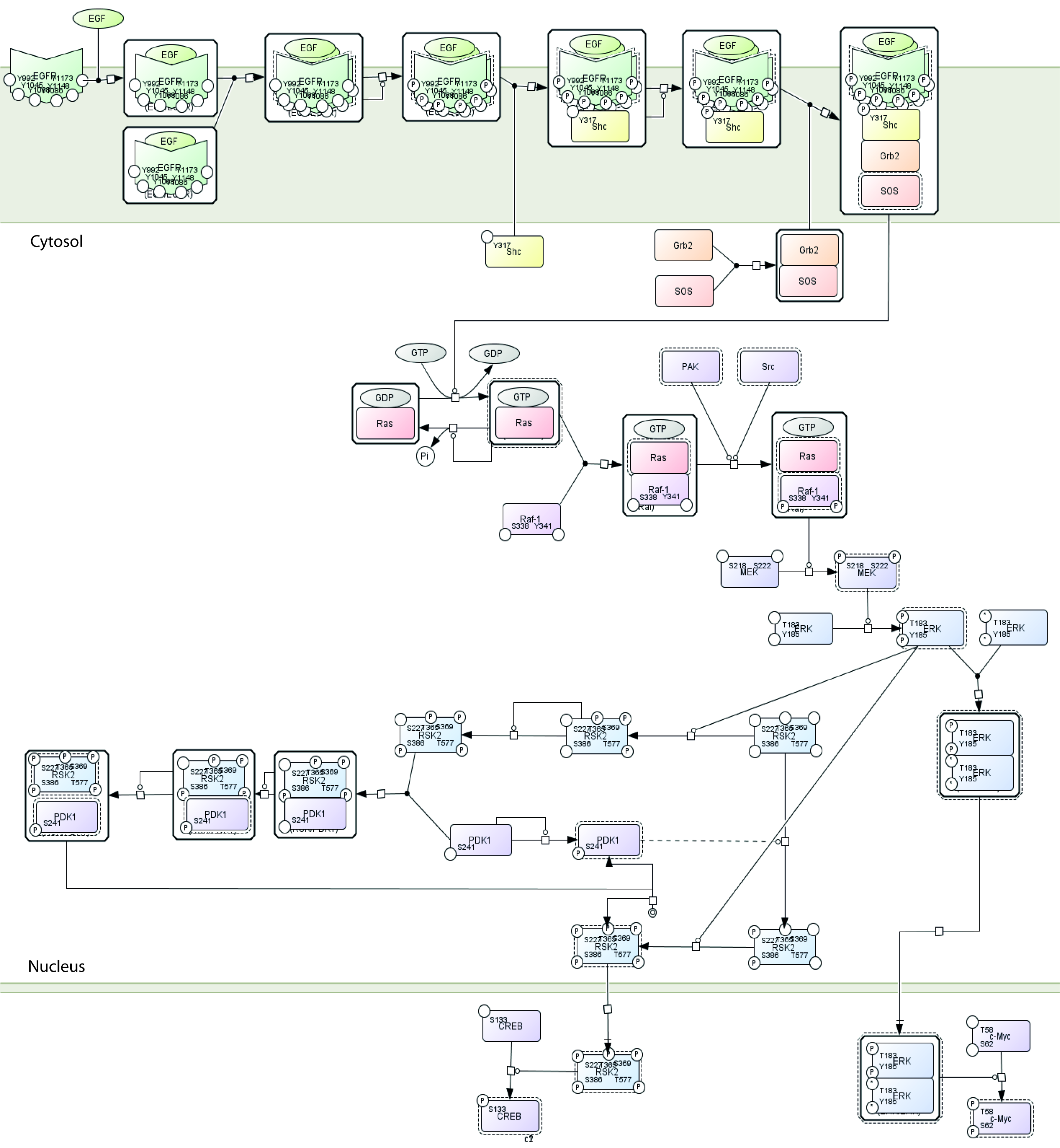

Supplement: Figure S4 — Simple EGFR signaling network edited by CellDesigner using SBGN (Systems Biology Graphical Notation). From the binding of EGF to EGFR on cell membrane to the catalysis of CREB and c-Myc within nucleus, there are 14 different proteins with 27 known reactions on the map. Upon recruitment of FGR-FGFR-Shc-Grb2-SOS complex, binding of GTP to Ras is induced, followed by formation of the GTP-Ras-Raf1 complex. Phosphorylation of the GTP-Ras-Raf1 complex is catalyzed by PAK and Src, leading to a series of subsequent phosphorylations of MEK, ERK and others. (TIF) [file pone.0083922.s004.tif]
