# Supplementary material for: Combining Machine Learning Systems and Multiple Docking Simulation Packages to Improve Docking Prediction Reliability for Network Pharmacology
Source: PLoS One. 2013 Dec 31;8(12):e83922. doi: 10.1371/journal.pone.0083922 (PMC3877102; doi:10.1371/journal.pone.0083922)
Supplement: Table S1 — Interaction types of the 36 interatomic contacts used in the development of both machine learning systems A and B. Contacts of atoms (C, N, O, F, P, S, Cl, Br and I) between the ligand and protein within a distance of 12 Å were counted. There were 81 different atom pairs, of which 45 were omitted in this study because none of PDBbind complexes contains F, P, Cl, Br or I atoms. As an example, C_C indicates the interaction type in which carbon atoms of a ligand interact with protein carbon atoms within a 12 Å radius. The number of occurrences of this interaction was counted. (DOCX) [file pone.0083922.s005.docx]

|  | Interatomic type |  |  | Interatomic type |
| --- | --- | --- | --- | --- |
| 1 | C_C |  | 19 | O_P |
| 2 | N_C |  | 20 | S_P |
| 3 | O_C |  | 21 | C_S |
| 4 | S_C |  | 22 | N_S |
| 5 | C_N |  | 23 | O_S |
| 6 | N_N |  | 24 | S_S |
| 7 | O_N |  | 25 | C_Cl |
| 8 | S_N |  | 26 | N_Cl |
| 9 | C_O |  | 27 | O_Cl |
| 10 | N_O |  | 28 | S_Cl |
| 11 | O_O |  | 29 | C_Br |
| 12 | S_O |  | 30 | N_Br |
| 13 | C_F |  | 31 | O_Br |
| 14 | N_F |  | 32 | S_Br |
| 15 | O_F |  | 33 | C_I |
| 16 | S_F |  | 34 | N_I |
| 17 | C_P |  | 35 | O_I |
| 18 | N_P |  | 36 | S_I |
