# Supplementary material for: Combining Machine Learning Systems and Multiple Docking Simulation Packages to Improve Docking Prediction Reliability for Network Pharmacology
Source: PLoS One. 2013 Dec 31;8(12):e83922. doi: 10.1371/journal.pone.0083922 (PMC3877102; doi:10.1371/journal.pone.0083922)
Supplement: Table S2 — Descriptions of the 74 molecular physicochemical properties used in the development of machine learning systems A and B. There were separated into six groups. These molecular properties were calculated using the Dragon software package (http://www.talete.mi.it/). (DOCX) [file pone.0083922.s006.docx]

| No. | Name | Description | Group |
| --- | --- | --- | --- |
| 1 | nCIC | number of rings (cyclomatic number) | Ring descriptors |
| 2 | nCIR | number of circuits | Ring descriptors |
| 3 | TRS | total ring size | Ring descriptors |
| 4 | Rperim | ring perimeter | Ring descriptors |
| 5 | Rbrid | ring bridge count | Ring descriptors |
| 6 | MCD | molecular cyclized degree | Ring descriptors |
| 7 | RFD | ring fusion density | Ring descriptors |
| 8 | RCI | ring complexity index | Ring descriptors |
| 9 | NRS | number of ring systems | Ring descriptors |
| 10 | NNRS | normalized number of ring systems | Ring descriptors |
| 11 | nR05 | number of 5-membered rings | Ring descriptors |
| 12 | nR06 | number of 6-membered rings | Ring descriptors |
| 13 | nR09 | number of 9-membered rings | Ring descriptors |
| 14 | nR10 | number of 10-membered rings | Ring descriptors |
| 15 | nBnz | number of benzene-like rings | Ring descriptors |
| 16 | ARR | aromatic ratio | Ring descriptors |
| 17 | D/Dtr03 | distance/detour ring index of order 3 | Ring descriptors |
| 18 | D/Dtr04 | distance/detour ring index of order 4 | Ring descriptors |
| 19 | D/Dtr05 | distance/detour ring index of order 5 | Ring descriptors |
| 20 | D/Dtr06 | distance/detour ring index of order 6 | Ring descriptors |
| 21 | D/Dtr07 | distance/detour ring index of order 7 | Ring descriptors |
| 22 | D/Dtr08 | distance/detour ring index of order 8 | Ring descriptors |
| 23 | D/Dtr09 | distance/detour ring index of order 9 | Ring descriptors |
| 24 | D/Dtr10 | distance/detour ring index of order 10 | Ring descriptors |
| 25 | D/Dtr11 | distance/detour ring index of order 11 | Ring descriptors |
| 26 | D/Dtr12 | distance/detour ring index of order 12 | Ring descriptors |
| 27 | Pol | polarity number | Topological indices |
| 28 | Wap | all-path Wiener index | Topological indices |
| 29 | PCR | ratio of multiple path count over path count | Walk and path counts |
| 30 | Wi_D | Wiener-like index from topological distance matrix (Wiener index) | 2D matrix-based descriptors |
| 31 | nCp | number of terminal primary C(sp3) | Functional group counts |
| 32 | nCs | number of total secondary C(sp3) | Functional group counts |
| 33 | nCt | number of total tertiary C(sp3) | Functional group counts |
| 34 | nCq | number of total quaternary C(sp3) | Functional group counts |
| 35 | nCrs | number of ring secondary C(sp3) | Functional group counts |
| 36 | nCrt | number of ring tertiary C(sp3) | Functional group counts |
| 37 | nCar | number of aromatic C(sp2) | Functional group counts |
| 38 | nCbH | number of unsubstituted benzene C(sp2) | Functional group counts |
| 39 | nCb- | number of substituted benzene C(sp2) | Functional group counts |
| 40 | nCconj | number of non-aromatic conjugated C(sp2) | Functional group counts |
| 41 | nR=Cs | number of aliphatic secondary C(sp2) | Functional group counts |
| 42 | nR=Ct | number of aliphatic tertiary C(sp2) | Functional group counts |
| 43 | nRCOOH | number of carboxylic acids (aliphatic) | Functional group counts |
| 44 | nArCOOH | number of carboxylic acids (aromatic) | Functional group counts |
| 45 | nRCONHR | number of secondary amides (aliphatic) | Functional group counts |
| 46 | nArCONHR | number of secondary amides (aromatic) | Functional group counts |
| 47 | nRCONR2 | number of tertiary amides (aliphatic) | Functional group counts |
| 48 | nCONN | number of urea (-thio) derivatives | Functional group counts |
| 49 | nN=C-N< | number of amidine derivatives | Functional group counts |
| 50 | nRNH2 | number of primary amines (aliphatic) | Functional group counts |
| 51 | nArNH2 | number of primary amines (aromatic) | Functional group counts |
| 52 | nRNHR | number of secondary amines (aliphatic) | Functional group counts |
| 53 | nRNR2 | number of tertiary amines (aliphatic) | Functional group counts |
| 54 | nN+ | number of positively charged N | Functional group counts |
| 55 | nROH | number of hydroxyl groups | Functional group counts |
| 56 | nArOH | number of aromatic hydroxyls | Functional group counts |
| 57 | nOHp | number of primary alcohols | Functional group counts |
| 58 | nOHs | number of secondary alcohols | Functional group counts |
| 59 | nROR | number of ethers (aliphatic) | Functional group counts |
| 60 | nArOR | number of ethers (aromatic) | Functional group counts |
| 61 | nSO2N | number of sulfonamides (thio-/dithio-) | Functional group counts |
| 62 | nPO4 | number of phosphates/thiophosphates | Functional group counts |
| 63 | nArX | number of X on aromatic ring | Functional group counts |
| 64 | nPyrrolidines | number of Pyrrolidines | Functional group counts |
| 65 | nOxolanes | number of Oxolanes | Functional group counts |
| 66 | nImidazoles | number of Imidazoles | Functional group counts |
| 67 | nPyridines | number of Pyridines | Functional group counts |
| 68 | nPyrimidines | number of Pyrimidines | Functional group counts |
| 69 | nHDon | number of donor atoms for H-bonds (N and O) | Functional group counts |
| 70 | nHAcc | number of acceptor atoms for H-bonds (N,O,F) | Functional group counts |
| 71 | nHBonds | number of intramolecular H-bonds (with N,O,F) | Functional group counts |
| 72 | Hy | hydrophilic factor | Molecular properties |
| 73 | TPSA(Tot) | topological polar surface area using N,O,S,P polar contributions | Molecular properties |
| 74 | ALOGP | Ghose-Crippen octanol-water partition coeff. (logP) | Molecular properties |
