# Supplementary material for: Combining Machine Learning Systems and Multiple Docking Simulation Packages to Improve Docking Prediction Reliability for Network Pharmacology
Source: PLoS One. 2013 Dec 31;8(12):e83922. doi: 10.1371/journal.pone.0083922 (PMC3877102; doi:10.1371/journal.pone.0083922)
Supplement: Table S3 — Compounds composing the training set for building the re-scoring function of machine learning system A. Chemical figures were obtained from PubChem website, and information about primary targets came from the work of Karaman et al.30. (DOCX) [file pone.0083922.s007.docx]

|  | Structure in 2D | Inhibitor | PubChem CID | Primary Targets |
| --- | --- | --- | --- | --- |
| 1 | 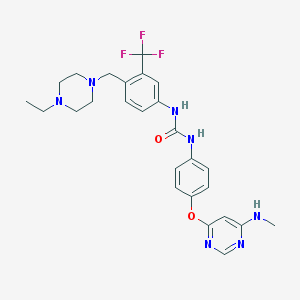 | AST_487 | 11409972 | FLT3  KIT |
| 2 | 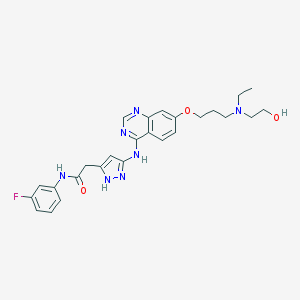 | AZD1152HQPA | 16007391 | AURKB |
| 3 | 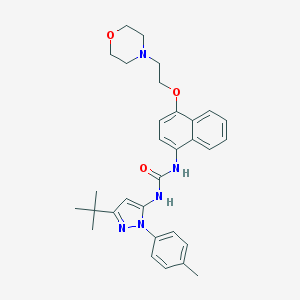 | BIRB_796 | 156422 | p38-alpha |
| 4 | 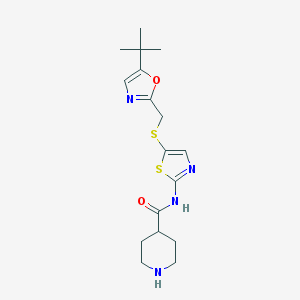 | BMS_387032 | 3025986 | CDK2 |
| 5 | 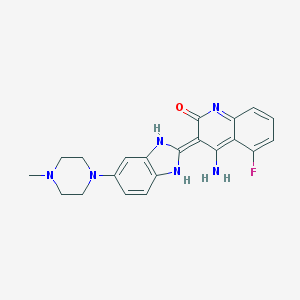 | CHIR_258 | 9886808 | FLT3  FGFR3 |
| 6 | 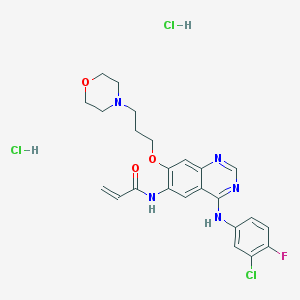 | CI_1033 | 156413 | EGFR  ERBB2 |
| 7 | 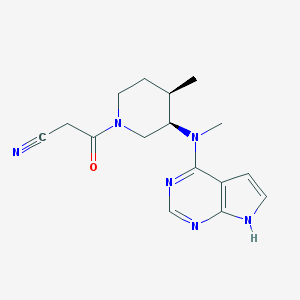 | CP_690550 | 9926791 | JAK3 |
| 8 | 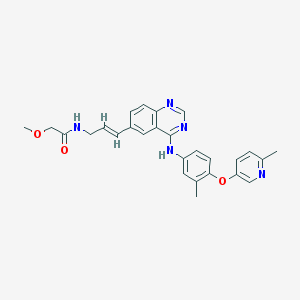 | CP_724714 | 9874913 | ERBB2 |
| 9 | 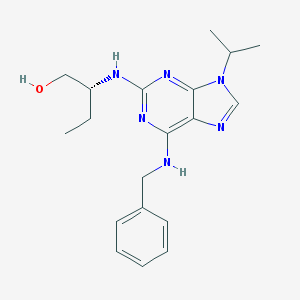 | CYC_202 /  Roscovitine | 160355 | CDK1  CDK2  CDK5 |
| 10 | 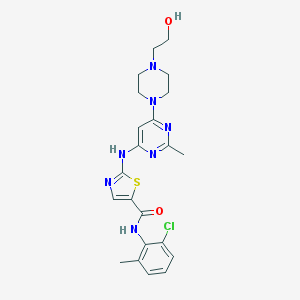 | Dasatinib | 3062316 | ABL1  SRC |
| 11 | 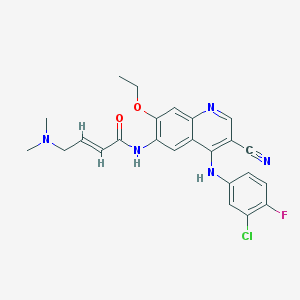 | EKB_569 | 6445562 | EGFR |
| 12 | 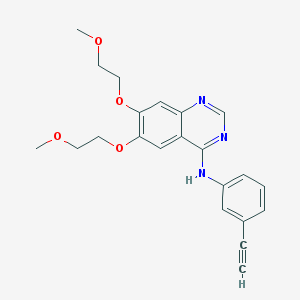 | Erlotinib | 176870 | EGFR |
| 13 | 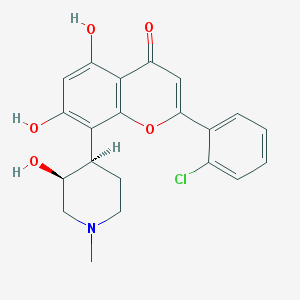 | Flavopiridol | 5287969 | CDK2  CDK9  other CDKs |
| 14 | 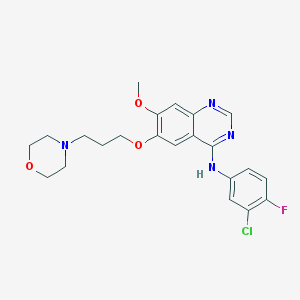 | Gefitinib | 123631 | EGFR |
| 15 | 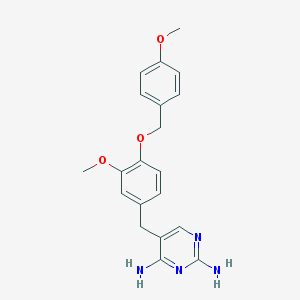 | GW_2580 | 11617559 | CSF1R |
| 16 | 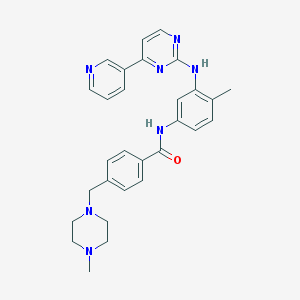 | Imatinib | 5291 | ABL1  KIT  PDGFRB |
| 17 | 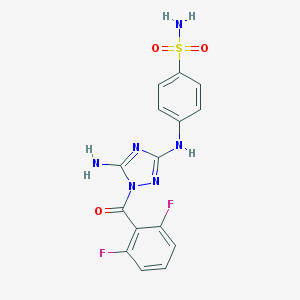 | JNJ_7706621 | 5330790 | CDK1  CDK2  AURKB |
| 18 | 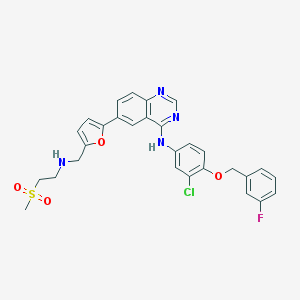 | Lapatinib | 208908 | EGFR  ERBB2 |
| 19 | 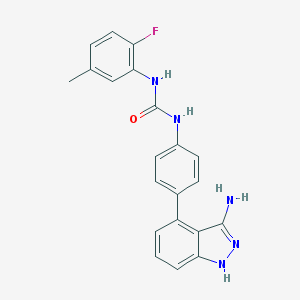 | Linifanib / ABT-869 | 11485656 | FLT3  CSF1R  VEGFR2 |
| 20 | 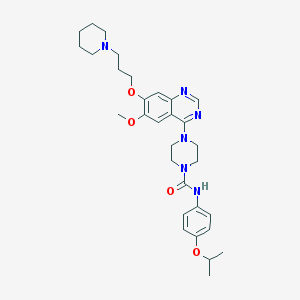 | MLN_518 | 3038522 | FLT3  KIT |
| 21 | 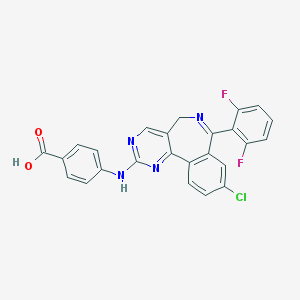 | MLN_8054 | 11712649 | AURKA |
| 22 | 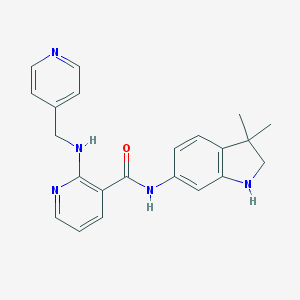 | Motesanib / AMG-706 | 11667893 | VEGFR2  FLT1  FLT4  KIT |
| 23 | 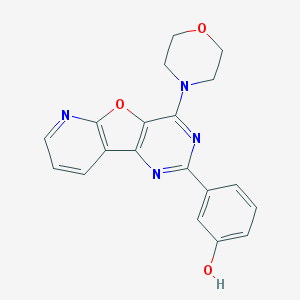 | PI_103 | 9884685 | PIK3CA |
| 24 | 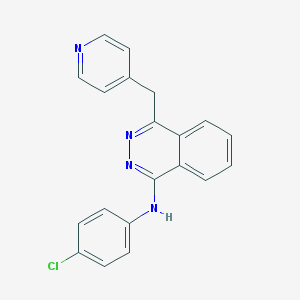 | PTK_787 | 151194 | VEGFR2 |
| 25 | 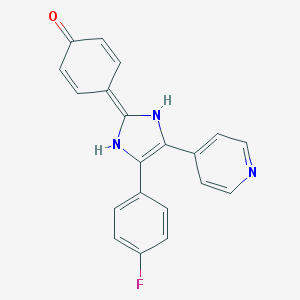 | SB_202190 | 5353940 | p38-alpha |
| 26 | 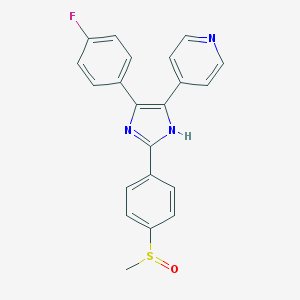 | SB_203580 | 176155 | p38-alpha |
| 27 | 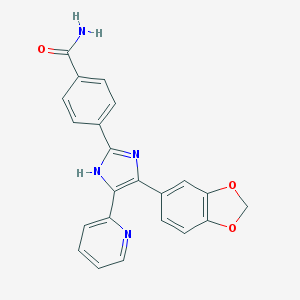 | SB_431542 | 4521392 | TGFBR1/ALK5  ACVR1B/ALK4 |
| 28 | 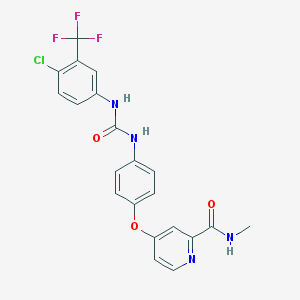 | Sorafenib | 216239 | VEGFR2  BRAF |
| 29 | 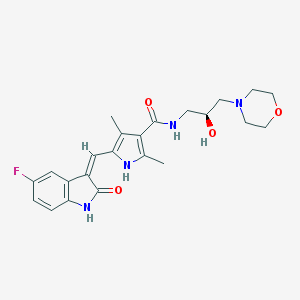 | SU_14813 | 10138259 | VEGFR2  FLT1  PDGFRB  KIT  FLT3 |
| 30 | 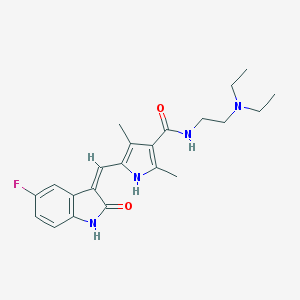 | Sunitinib | 5329102 | KIT  VEGFR2  FLT3 |
| 31 | 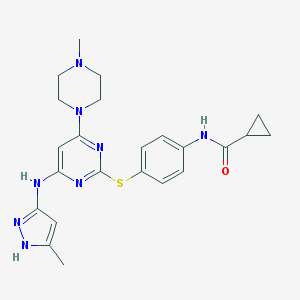 | VX_680 | 5494449 | AURKA  AURKB  AURKC |
| 32 | 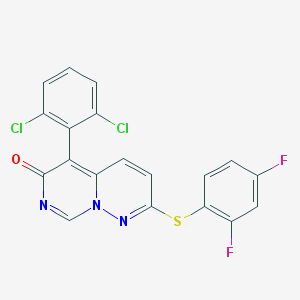 | VX_745 | 3038525 | p38-alpha |
| 33 | 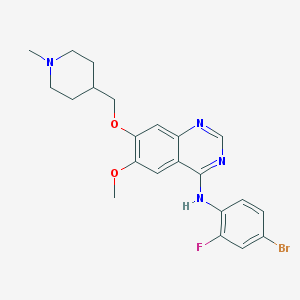 | ZD_6474 | 3081361 | VEGFR2  EGFR  RET |
