# Supplementary material for: Combining Machine Learning Systems and Multiple Docking Simulation Packages to Improve Docking Prediction Reliability for Network Pharmacology
Source: PLoS One. 2013 Dec 31;8(12):e83922. doi: 10.1371/journal.pone.0083922 (PMC3877102; doi:10.1371/journal.pone.0083922)
Supplement: Table S4 — Proteins for generating binding modes that composed the training set comprising the re-scoring function of machine learning system A. (DOCX) [file pone.0083922.s008.docx]

|  | Protein Name | PDB ID | Kinase Group |  |  | Protein Name | PDB ID | Kinase Group |
| --- | --- | --- | --- | --- | --- | --- | --- | --- |
| 1 | ABL1 | 2G1T | TK |  | 74 | JNK1 | 3PZE | CMGC |
| 2 | ABL1(T315I) | 2V7A | TK |  | 75 | JNK2 | 3NPC | CMGC |
| 3 | ABL2 | 3GVU | TK |  | 76 | JNK3 | 2B1P | CMGC |
| 4 | ACVR1(ALK2) | 3OOM | TKL |  | 77 | KIT | 3G0E | TK |
| 5 | ACVR2A(ActR2) | 3SOC | TKL |  | 78 | LCK | 2OFV | TK |
| 6 | ACVR2B | 2QLU | TKL |  | 79 | LIMK1 | 3S95 | TKL |
| 7 | ACVRL1(ALK1) | 3MY0 | TKL |  | 80 | LKB1 | 2WTK | CAMK |
| 8 | AKT1 | 4EJN | AGC |  | 81 | LOK | 4BC6 | STE |
| 9 | AKT2 | 2X39 | AGC |  | 82 | LYN | 3A4O | TK |
| 10 | ALK | 3AOX | TK |  | 83 | MAP3K5 | 2CLQ | STE |
| 11 | AMPK-alpha1(AMPKA1) | 2V8Q | CAMK |  | 84 | MAPKAPK2 | 3M2W | CAMK |
| 12 | AMPK-alpha2(AMPKA2) | 3AQV | CAMK |  | 85 | MEK1(MAP2K1) | 3EQD | STE |
| 13 | AURKA | 3DJ5 | Other |  | 86 | MEK2(MAP2K2) | 1S9I | STE |
| 14 | AURKB(AurB) | 4AF3 | Other |  | 87 | MEK4(MAP2K4) | 3ALN | STE |
| 15 | BMPR2 | 3G2F | TKL |  | 88 | MEK6(MAP2K6) | 3FME | STE |
| 16 | BMX(EtK) | 3SXS | TK |  | 89 | MERTK | 2P0C | TK |
| 17 | BRAF | 4E26 | TKL |  | 90 | MET | 1R0P | TK |
| 18 | BRAF(V600E) | 4FK3 | TKL |  | 91 | MKNK1 | 2Y9Q | CAMK |
| 19 | BTK | 3PJ2 | TK |  | 92 | MKNK2 | 3VN9 | CAMK |
| 20 | CAMK1G(CaMK1-gamma) | 2JAM | CAMK |  | 93 | MLK1 | 3DTC | TKL |
| 21 | CAMK2A(CaMK2-alpha) | 2VZ6 | CAMK |  | 94 | MRCKA | 4AW2 | AGC |
| 22 | CAMK2B(CaMK2-beta) | 3BHH | CAMK |  | 95 | MRCKB | 3TKU | AGC |
| 23 | CAMK2D(CaMK2-delta) | 2VN9 | CAMK |  | 96 | MST3(STK24) | 3CKX | STE |
| 24 | CAMK2G(CaMK2-gamma) | 2V7O | CAMK |  | 97 | MST4 | 3GGF | STE |
| 25 | CAMKK2 | 2ZV2 | Other |  | 98 | NEK1 | 4B9D | Other |
| 26 | CDK2 | 3RM6 | CMGC |  | 99 | NEK2 | 2XNM | Other |
| 27 | CDK5 | 1UNH | CMGC |  | 100 | NEK7 | 2WQN | Other |
| 28 | CDK7 | 1UA2 | CMGC |  | 101 | p38-alpha | 3ZS5 | CMGC |
| 29 | CDK8 | 3RGF | CMGC |  | 102 | p38-beta | 3GC8 | CMGC |
| 30 | CDK9 | 3MY1 | CMGC |  | 103 | p38-gamma | 1CM8 | CMGC |
| 31 | CHEK1(Chk1) | 3PA3 | CAMK |  | 104 | PAK1 | 2HY8 | STE |
| 32 | CLK1 | 1Z57 | CMGC |  | 105 | PAK4 | 2X4Z | STE |
| 33 | CLK2 | 3NR9 | CMGC |  | 106 | PAK6 | 2ODB | STE |
| 34 | CLK3 | 2WU7 | CMGC |  | 107 | PAK7 | 2F57 | STE |
| 35 | CSF1R(CSFR) | 2I0Y | TK |  | 108 | PDK1 | 2BIY | AGC |
| 36 | CSK | 1BYG | TK |  | 109 | PHKG1 | 1PHK | CAMK |
| 37 | CSNK1D(CK1-D) | 3UZP | CK1 |  | 110 | PHKG2 | 2Y7J | CAMK |
| 38 | CSNK1G1(CK1-G1) | 2CMW | CK1 |  | 111 | PIM1 | 3R04 | CAMK |
| 39 | CSNK1G2(CK1-G2) | 2C47 | CK1 |  | 112 | PIM2 | 2IWI | CAMK |
| 40 | CSNK1G3(CK1-G3) | 2IZU | CK1 |  | 113 | PKAC-alpha(PKACa) | 3POO | AGC |
| 41 | CSNK2A1(CK2-A1) | 3BQC | Other |  | 114 | PKN1 | 1CXZ | AGC |
| 42 | CSNK2A2(CK2-A2) | 3OFM | Other |  | 115 | PLK1 | 2OWB | Other |
| 43 | CaMK4 | 2W4O | CAMK |  | 116 | PLK4 | 3COK | Other |
| 44 | DAPK1 | 3GU4 | CAMK |  | 117 | PRKCH | 3TXO | AGC |
| 45 | DAPK2 | 2CKE | CAMK |  | 118 | PRKCQ | 2JED | AGC |
| 46 | DAPK3 | 3BQR | CAMK |  | 119 | PRKR | 2A19 | Other |
| 47 | DMPK | 2VD5 | AGC |  | 120 | PTK2 | 4EBV | TK |
| 48 | DRAK2 | 3LM5 | CAMK |  | 121 | PTK2B | 3FZT | TK |
| 49 | EGFR | 2RGP | TK |  | 122 | RAF1 | 3KUC | TKL |
| 50 | EGFR(G719S) | 2ITN | TK |  | 123 | RET | 2IVS | TK |
| 51 | EGFR(L858R) | 2ITV | TK |  | 124 | RPS6KA1 | 2Z7R | AGC |
| 52 | EPHA2 | 1MQB | TK |  | 125 | RPS6KA3 | 4D9U | AGC |
| 53 | EPHA3(EphA3) | 2QO9 | TK |  | 126 | RPS6KA5 | 3KN5 | AGC |
| 54 | EPHA5(EphA5) | 2RAP | TK |  | 127 | Sgk085 | 2X4F | CAMK |
| 55 | EPHA7(EphA7) | 3DKO | TK |  | 128 | SLK | 2J51 | STE |
| 56 | EPHB4 | 2VWY | TK |  | 129 | SRC | 1Y57 | TK |
| 57 | ERBB2(HER2) | 3PP0 | TK |  | 130 | SRPK1 | 1WBP | CMGC |
| 58 | ERBB4 | 2R4B | TK |  | 131 | STK16 | 2BUJ | Other |
| 59 | ERK1 | 2ZOQ | CMGC |  | 132 | SYK | 4DFL | TK |
| 60 | ERK2 | 2OJG | CMGC |  | 133 | TGFBR1 | 3TZM | TKL |
| 61 | ERK5 | 4B99 | CMGC |  | 134 | TIE2 | 2P4I | TK |
| 62 | FES | 4E93 | TK |  | 135 | TNIK | 2X7F | STE |
| 63 | FGFR1 | 3RHX | TK |  | 136 | TNK2 | 1U4D | TK |
| 64 | FGFR2 | 3RI1 | TK |  | 137 | TRKA | 4AOJ | TK |
| 65 | FLT1(VEGFR-1) | 3HNG | TK |  | 138 | TRKB | 4AT5 | TK |
| 66 | GSK3B | 1Q5K | CMGC |  | 139 | TRKC | 3V5Q | TK |
| 67 | HCK | 2HK5 | TK |  | 140 | TTK | 3HMO | Other |
| 68 | IGF1R | 2OJ9 | TK |  | 141 | TYK2 | 3LXN | TK |
| 69 | INSR | 3EKN | TK |  | 142 | VEGFR2 | 3VHE | TK |
| 70 | ITK | 3MIY | TK |  | 143 | WEE1 | 2IN6 | Other |
| 71 | JAK1 | 3EYG | TK |  | 144 | YSK1 | 2XIK | STE |
| 72 | JAK2 | 4FVQ | TK |  | 145 | ZAP70 | 1U59 | TK |
| 73 | JAK3 | 3LXK | TK |  |  |  |  |  |
