# Supplementary material for: Combining Machine Learning Systems and Multiple Docking Simulation Packages to Improve Docking Prediction Reliability for Network Pharmacology
Source: PLoS One. 2013 Dec 31;8(12):e83922. doi: 10.1371/journal.pone.0083922 (PMC3877102; doi:10.1371/journal.pone.0083922)
Supplement: Table S5 — Kinase proteins for the case study using the screening approach proposed in present work. There were 139 different kinase structures covering eight kinase groups in total. (DOCX) [file pone.0083922.s009.docx]

|  | Protein Name | PDB ID | Kinase Group |  |  | Protein Name | PDB ID | Kinase Group |
| --- | --- | --- | --- | --- | --- | --- | --- | --- |
| 1 | ABL1 | 2HZI | TK |  | 71 | KIT | 1T46 | TK |
| 2 | ABL2 | 3HMI | TK |  | 72 | LCK | 3MPM | TK |
| 3 | ACVR1(ALK2) | 3Q4U | TKL |  | 73 | LIMK1 | 3S95 | TKL |
| 4 | ACVR2A(ActR2) | 3Q4T | TKL |  | 74 | LKB1 | 2WTK | CAMK |
| 5 | ACVR2B | 2QLU | TKL |  | 75 | LOK | 2J7T | STE |
| 6 | AKT1 | 4EJN | AGC |  | 76 | LYN | 3A4O | TK |
| 7 | AKT2 | 1O6L | AGC |  | 77 | MAP3K5 | 2CLQ | STE |
| 8 | ALK | 4FOC | TK |  | 78 | MAPKAPK2 | 3M2W | CAMK |
| 9 | AMPK-alpha1(AMPKA1) | 2V8Q | CAMK |  | 79 | MEK1(MAP2K1) | 3EQI | STE |
| 10 | AMPK-alpha2(AMPKA2) | 3AQV | CAMK |  | 80 | MEK2(MAP2K2) | 1S9I | STE |
| 11 | AURKA | 3DJ6 | Other |  | 81 | MEK4(MAP2K4) | 3ALN | STE |
| 12 | AURKB(AurB) | 4AF3 | Other |  | 82 | MEK6(MAP2K6) | 3FME | STE |
| 13 | BMPR2 | 3G2F | TKL |  | 83 | MERTK | 3BRB | TK |
| 14 | BMX(EtK) | 3SXR | TK |  | 84 | MET | 3F66 | TK |
| 15 | BRAF | 4E26 | TKL |  | 85 | MKNK1 | 2Y9Q | CAMK |
| 16 | BTK | 3GEN | TK |  | 86 | MKNK2 | 2HW7 | CAMK |
| 17 | CAMK1G(CaMK1-gamma) | 2JAM | CAMK |  | 87 | MLK1 | 3DTC | TKL |
| 18 | CAMK2A(CaMK2-alpha) | 2VZ6 | CAMK |  | 88 | MRCKA | 4AW2 | AGC |
| 19 | CAMK2B(CaMK2-beta) | 3BHH | CAMK |  | 89 | MRCKB | 3TKU | AGC |
| 20 | CAMK2D(CaMK2-delta) | 2WEL | CAMK |  | 90 | MST3(STK24) | 3CKX | STE |
| 21 | CAMK2G(CaMK2-gamma) | 2V7O | CAMK |  | 91 | MST4 | 3GGF | STE |
| 22 | CaMK4 | 2W4O | CAMK |  | 92 | NEK1 | 4B9D | Other |
| 23 | CAMKK2 | 2ZV2 | Other |  | 93 | NEK2 | 2W5A | Other |
| 24 | CDK2 | 1JVP | CMGC |  | 94 | NEK7 | 2WQN | Other |
| 25 | CDK5 | 1UNG | CMGC |  | 95 | p38-alpha | 3LFF | CMGC |
| 26 | CDK7 | 1UA2 | CMGC |  | 96 | p38-beta | 3GP0 | CMGC |
| 27 | CDK8 | 3RGF | CMGC |  | 97 | p38-gamma | 1CM8 | CMGC |
| 28 | CDK9 | 3BLR | CMGC |  | 98 | PAK1 | 3Q4Z | STE |
| 29 | CHEK1(Chk1) | 2YEX | CAMK |  | 99 | PAK4 | 2CDZ | STE |
| 30 | CLK1 | 2VAG | CMGC |  | 100 | PAK6 | 2ODB | STE |
| 31 | CLK2 | 3NR9 | CMGC |  | 101 | PAK7 | 2F57 | STE |
| 32 | CLK3 | 2WU6 | CMGC |  | 102 | PDK1 | 3NAX | AGC |
| 33 | CSF1R(CSFR) | 2I1M | TK |  | 103 | PHKG1 | 1PHK | CAMK |
| 34 | CSK | 1BYG | TK |  | 104 | PHKG2 | 2Y7J | CAMK |
| 35 | CSNK1D(CK1-D) | 3UYT | CK1 |  | 105 | PIM1 | 3A99 | CAMK |
| 36 | CSNK1G1(CK1-G1) | 2CMW | CK1 |  | 106 | PIM2 | 2IWI | CAMK |
| 37 | CSNK1G2(CK1-G2) | 2C47 | CK1 |  | 107 | PKAC-alpha(PKACa) | 3OVV | AGC |
| 38 | CSNK1G3(CK1-G3) | 2IZR | CK1 |  | 108 | PKN1 | 1CXZ | AGC |
| 39 | CSNK2A1(CK2-A1) | 3NSZ | Other |  | 109 | PLK1 | 2RKU | Other |
| 40 | CSNK2A2(CK2-A2) | 3OFM | Other |  | 110 | PLK4 | 3COK | Other |
| 41 | DAPK1 | 2W4J | CAMK |  | 111 | PRKCH | 3TXO | AGC |
| 42 | DAPK2 | 2CKE | CAMK |  | 112 | PRKCQ | 1XJD | AGC |
| 43 | DAPK3 | 3BHY | CAMK |  | 113 | PRKR | 2A19 | Other |
| 44 | DMPK | 2VD5 | AGC |  | 114 | PTK2 | 1MP8 | TK |
| 45 | DRAK2 | 3LM0 | CAMK |  | 115 | PTK2B | 3FZS | TK |
| 46 | EGFR | 3POZ | TK |  | 116 | RAF1 | 1C1Y | TKL |
| 47 | EPHA2 | 1MQB | TK |  | 117 | RET | 2IVS | TK |
| 48 | EPHA3(EphA3) | 2QO9 | TK |  | 118 | RPS6KA1 | 2Z7Q | AGC |
| 49 | EPHA5(EphA5) | 2RAP | TK |  | 119 | RPS6KA3 | 4D9T | AGC |
| 50 | EPHA7(EphA7) | 3DKO | TK |  | 120 | RPS6KA5 | 3KN5 | AGC |
| 51 | EPHB4 | 2VWX | TK |  | 121 | Sgk085 | 2X4F | CAMK |
| 52 | ERBB2(HER2) | 3PP0 | TK |  | 122 | SLK | 2J51 | STE |
| 53 | ERBB4 | 2R4B | TK |  | 123 | SRC | 3U4W | TK |
| 54 | ERK1 | 2ZOQ | CMGC |  | 124 | SRPK1 | 1WBP | CMGC |
| 55 | ERK5 | 4B99 | CMGC |  | 125 | STK16 | 2BUJ | Other |
| 56 | FES | 3BKB | TK |  | 126 | SYK | 1XBB | TK |
| 57 | FGFR1 | 3DPK | TK |  | 127 | TGFBR1 | 3HMM | TKL |
| 58 | FGFR2 | 3B2T | TK |  | 128 | TIE2 | 2OO8 | TK |
| 59 | FLT1(VEGFR-1) | 3HNG | TK |  | 129 | TNIK | 2X7F | STE |
| 60 | GSK3B | 1J1B | CMGC |  | 130 | TNK2 | 3EQR | TK |
| 61 | HCK | 2HK5 | TK |  | 131 | TRKA | 4AOJ | TK |
| 62 | IGF1R | 3LW0 | TK |  | 132 | TRKB | 4AT4 | TK |
| 63 | INSR | 3EKK | TK |  | 133 | TRKC | 3V5Q | TK |
| 64 | ITK | 3T9T | TK |  | 134 | TTK | 3HMP | Other |
| 65 | JAK1 | 4E5W | TK |  | 135 | TYK2 | 3NZ0 | TK |
| 66 | JAK2 | 3UGC | TK |  | 136 | VEGFR2 | 2XIR | TK |
| 67 | JAK3 | 3LXL | TK |  | 137 | WEE1 | 1X8B | Other |
| 68 | JNK1 | 3ELJ | CMGC |  | 138 | YSK1 | 2XIK | STE |
| 69 | JNK2 | 3NPC | CMGC |  | 139 | ZAP70 | 1U59 | TK |
| 70 | JNK3 | 3OY1 | CMGC |  |  |  |  |  |
