# Supplementary material for: Combining Machine Learning Systems and Multiple Docking Simulation Packages to Improve Docking Prediction Reliability for Network Pharmacology
Source: PLoS One. 2013 Dec 31;8(12):e83922. doi: 10.1371/journal.pone.0083922 (PMC3877102; doi:10.1371/journal.pone.0083922)
Supplement: Table S7 — Parameters and settings for the docking simulation in this work. (DOCX) [file pone.0083922.s011.docx]

|  | Program | Version | Docking algorithm and parameters |
| --- | --- | --- | --- |
| 1. | eHiTS | 2012 | - Docking algorithm is a fragment-based incremental method that cleaves the test compound into pieces then docks those to the binding site in an incremental fashion. - Active site based on the ligand position and residue atoms are selected within 10Å. - Output 1000 top-ranked solutions. - Accuracy level 6 (top level). |
| 2. | GOLD | 5.1 | - Uses a genetic algorithm to generate conformers in a binding site. - Active site based on the ligand position and residue atoms are selected within 10Å. - GA runs is 50 along with 200% search efficiency. - Flips pyramidal N, amide bonds, ring corners, all planar R-NR1R2 and protonated carboxylic acids. - Output maximum 300 top-ranked solutions. |
| 3. | AutoDock Vina | 1.1.2 | - Adopts a gradient-based local search algorithm for seeking a local optimum. - Active site based on the center of the ligand position and residue atoms are selected within 10Å. - Maximum 1000 of binding modes to generate. - Maximum energy difference between the best binding mode and the worst one displayed is 10 (kcal/mol). |
